# Is Migraine an MPV-Related Disease? An Observational Study of Polish Neurological Patients

**DOI:** 10.1155/2019/9454580

**Published:** 2019-12-06

**Authors:** Katarzyna Brzeźniakiewicz-Janus, Marcus D. Lancé, Andrzej Tukiendorf, Joanna Rupa-Matysek, Sybilla Brzozowska-Mańkowska, Mirosław Franków, Edyta Wolny-Rokicka, Lidia Gil

**Affiliations:** ^1^Department of Haematology, Faculty of Medicine and Health Science, University of Zielona Góra, Zielona Góra, Poland; ^2^Department of Anesthesiology, Intensive Care Unit and Perioperative Medicine, Hamad Medical Corporation, Doha, Qatar; ^3^Department of Public Health, Wrocław Medical University, Wrocław, Poland; ^4^Department of Haematology and Bone Marrow Transplantation, Poznań University of Medical Sciences, Poznań, Poland; ^5^Emergency Department, Faculty of Medicine and Health Science University of Zielona Góra, Zielona Góra, Poland; ^6^Department of Surgery and Oncology, Faculty of Medicine and Health Science, University of Zielona Góra, Zielona Góra, Poland

## Abstract

Many studies have found correlations between abnormal MPV and clinical reactivity in a variety of diseases. In the present paper, we sought MPV-related neurological diseases that are less frequently reported in the literature. The electronic medical records of 852 neurological patients with mean platelet volume (MPV) measurements (*F* = 45%, *age* = 55.7 ± 18.7, 8–104) were searched after the patients had received a diagnosis of a neurological disease (new and old episodes) according to the nine classes of the International Statistical Classification of Diseases and Related Health Problems, 10th revision (ICD-10). A set of consecutive statistical methods (i.e., cluster analysis, segmented regression, linear correlation, propensity score matching, and mixed effects Poisson regression) were used to establish a link between MPV and neurological disease. A statistically significant (*p* < 0.05) relationship with MPV was found only in pain syndrome patients, with seven out of eight clinically diagnosed migraine episodes. With all other ICD-10 classes of neurological diseases, the effect of MPV was found to be nonsignificant (*p* > 0.05). MPV may implicate a clinical relationship with pain syndrome and migraine episodes. More complex statistics could help analyse data and find new correlations.

## 1. Introduction

The mean platelet volume (MPV) is an easily measurable haematologic standard parameter. Many scientists have discussed its interpretation and have found correlations between abnormal MPV and clinical reactivity in a variety of diseases [1]. It is believed that several diseases are associated with MPV changes. In addition, abnormal MPV is associated with poor prognosis in several diseases (also confirmed in our study [2]). Large platelets are associated with prothrombotic states and cardiovascular disease, whereas small platelets are detected in chronic inflammatory diseases or rheumatoid arthritis [3]. However, it is not difficult to find opposite results in the literature. For example, in some studies, there was no statistically significant association between appendicitis and MPV [4–6], while in another, the MPV was significantly lower in acute gangrenous appendicitis than in control healthy subjects [7]. However, the MPV is elevated in rheumatoid arthritis, and its cut-off level was estimated to be 10.4 fL [8]. Thus, the interpretation of the MPV is not as straightforward as it might appear [1]. One reason for this might be that there is currently no preanalytical standard when dealing with MPV measurements [9]. In addition, some authors have even demonstrated the high technical diversity of MPV laboratory measurement. Nevertheless, after MPV measurement became fully automated, in the past decades, it has become the subject of investigation in thousands of publications [1].

In the present paper, we sought MPV-related neurological diseases that are less frequently reported in the literature. Some data suggest the role of changes in platelet functions among patients with migraine [10], particularly with regard to aura and its impact on vascular events and stroke [11, 12]. Therefore, the question arises whether MPV, as a marker of platelet activation, is directly or indirectly connected with migraine attacks and if there is any effect on other vascular events.

It was assumed that the effect of MPV on a neurological disease should be more explicit in some “nonspecific” clinical conditions, particularly those which cooccur with each other. If this relationship could be found, the identification of MPV-related diseases could be more feasible.

## 2. Materials

Altogether, 958 individual MPV measurements in 852 patients (*F* = 45%, *aged* = 55.7 ± 18.7, 8–104) were assessed after the diagnosis of a neurologic disease (new and old episodes) according to the ICD-10 at the Gorzów Wlkp. Multi-Specialist Hospital in the one and a half-year period from 1 January 2017 to 30 June 2018 (repeated MPV values from an individual patient were averaged).

Statistics of the patients diagnosed with neurological disorders according to ICD-10 class are reported in Table 1.

In the meningitis group, diagnoses were made regarding infection of a brain structure with a bacterial or of undetermined aetiology. Alzheimer's dementias, recognized as Alzheimer's disease, Parkinson's disease, dystonia, and extrapyramidal disorders were collected in the “neurodegenerative disease” group. Multiple sclerosis is included as a separate group. The epilepsy group includes both primary and secondary epileptic episodes from head trauma, psychoactive substance use, electrolyte disturbances, CNS infections, or neoplastic diseases. The pain syndrome group includes migraine headaches of vascular origin or undetermined aetiology. Ischaemic syndromes included transient ischaemic episodes, stroke syndromes, and one diagnosis of pathological hypersomnia. In the neuralgia and peripheral nerve palsy group, facial, peripheral, and nerve root compression syndromes, neuropathies of various aetiologies, and one case of Guillain-Barré's syndrome were reported. Paralysis syndromes include myopathic myopathies; mono-, di-, and tetraplegias; infantile paralysis; myasthenia gravis; and infections of unknown cause. The last group includes diagnoses such as hydrocephalus, cerebral oedema, brain disorders, and encephalopathy with an undetermined cause.

Measurement of the MPV blood marker was performed in the hospital Laboratory Unit using Sysmex XN-2000 (Sysmex Corporation, Japan) analytical systems with EDTA-KE/2.7 mL samples.

## 3. Methods and Results

**Figure 1:**
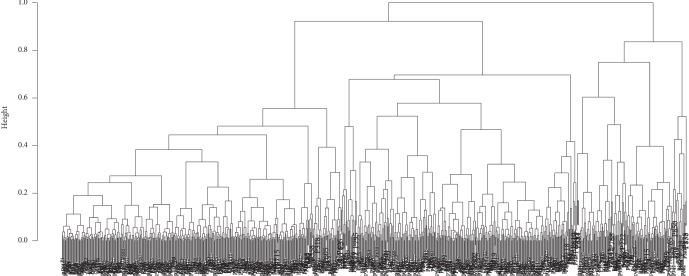
Classification tree of patients according to age, sex, and MPV (*n* = 852). Particular patients (coded by “P” numbers) from up to down are hierarchically aggregated in separated branches, ultimately representing individual leaves in the dendrogram; based on this, “families of patients” can be distinguished for the assumed height of pruning.

To determine a possible effect of MPV on the analysed neurologic diseases, taxonomic statistics were first assessed in the statistical analysis (an analogous method was performed in [13, 14]). In this approach, the patients' sexes, ages, and MPV measurements were used to classify the subjects. An original metric (distance) proposed by Marczewski and Steinhaus [15], which relies on the use of a symmetric difference between objects, was then applied. In short, the taxonomic distance (*D*) between objects (*A*, *B*) is defined as follows: *D* = |*A*‐*B*|/max(*A*, *B*), where the nominator is the modulus of *A*‐*B* and the denominator is the maximum of *A* and *B*. Before performing the distance calculation, the data were normalized into the 0–1 range. The resulting classification tree is presented in Figure 1.

**Figure 2:**
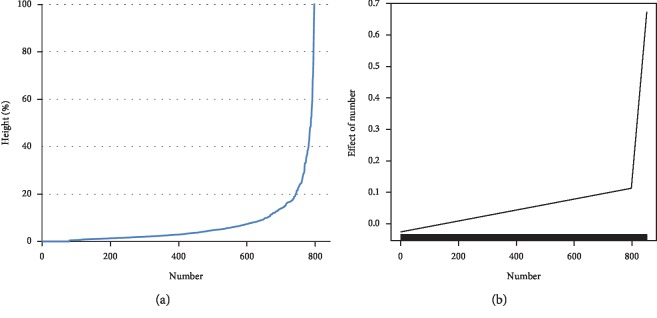
Scree plot of observed heights and the fitted segmented regression (*n* = 852).

By arranging the taxonomic heights (distances), it can be seen that until a certain order number, the heights are relatively similar to each other; above this number, the height rapidly increases (see Figure 2(a)). Using segmented regression [16], the threshold value for the scree can be estimated (see fitted segmented regression in Figure 2(b)).

It can be seen from the segmented regression in Figure 2(b) that the *estimated* *breakpoint* *number* = 799. The classification tree shown in Figure 1 was pruned at the resulting breakpoint height of 0.2158. In this way, 852‐799 = 53 clusters of patients identified as representing unique disease cases (the clusters were enumerated; enumeration was arbitrary and only had a technical/analytical meaning).

Then, to look for a closer similarity between the clusters, first, the percentages of patients in each ICD-10 disease class were correlated with each other using Pearson's *r* linear coefficient. A pair of clusters was considered similar if the estimated *p* value of their correlation was <0.05.

**Figure 3:**
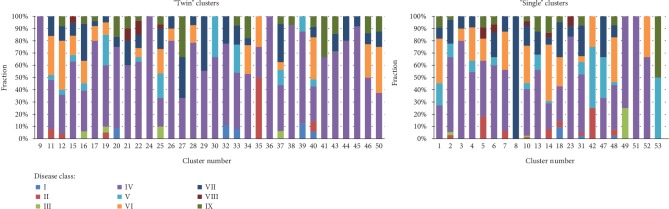
Fractions of diseases in “twin” (*n* = 456 patients) and “single” clusters (*n* = 396).

Then, clusters were divided into “twin” = similar and “single” = dissimilar collections of patients following the fractions of the analysed diseases. To determine whether clusters were “twin” or “single,” patient cluster matching was performed using propensity score matching [17]. In this way, 32 clusters with 456 patients (9, 11, 12, 15, 16, 17, 19, 20, 21, 22, 24, 25, 26, 27, 28, 29, 30, 32, 33, 34, 35, 36, 37, 38, 39, 40, 41, 43, 44, 45, 46, and 50) were established as “twin” clusters, and 21 with 396 patients (1, 2, 3, 4, 5, 6, 7, 8, 10, 13, 14, 18, 23, 31, 42, 47, 48, 49, 51, 52, and 53) were established as “single” clusters. The graphical characteristics of the analysed diseases in the “twin” and “single” clusters are presented in Figure 3.

Roughly, Figure 3 indicates that “twin” patterns of columns are less mosaic (more “stable”) than the (more “dynamic”) “single” fractions of the diseases, and the picture justifies the chosen statistical methodology.

For the established “twin” and “single” clusters of patients, the effects of sex, age, and MPV on the number of disease cases were estimated using mixed effects Poisson regression [18]. It is of note that a statistically significant (*p* < 0.05) result for MPV was found only in 24 “single” patients (12 clusters: 1, 2, 4, 6, 10, 13, 14, 31, 42, 47, 48, and 53) of the total 50 pain syndrome subjects, while in the remaining 26 “twin” pain syndrome patients (12 clusters: 11, 12, 15, 16, 19, 22, 25, 30, 33, 37, 39, and 40), the effect of MPV was nonsignificant. The relative risk estimates for the analysed triplet together with other statistically significant selected biomarkers (red blood cells (RBCs), haematocrit, and haemoglobin) for “twin” and “single” clusters are reported in Table 2 (the authors discovered a wide range of additional selected clinical biomarkers that were also used in the statistical analysis).

Table 2 shows a statistically significant effect of female gender on the risk of suffering from a pain syndrome; however, the impact of age on patients can be ignored in both the “twin” and “single” clusters. As mentioned above, MPV and the other analysed biomarkers (RBC, haematocrit, and haemoglobin) can be used to predict a relative risk but only in “single” patients, because the risk for “twin” subjects is statistically nonsignificant. Furthermore, for the results reported for MPV-related pain syndrome, if the difference in the biomarker's concentration is 10 fL, then the relative risk for the disease increases up to (1.03^10^‐1)∗100% = 34% (one-third). However, in MPV-related patients, an opposite effect of RBC, haematocrit, and haemoglobin on the number of pain syndrome cases was observed, i.e., the higher the levels of biomarkers, the lower the chance of a pain syndrome.

Additionally, for identified “twin” and “single” (i.e., MPV-unrelated and MPV-related) patients suffering from a pain syndrome, differences in other biomarkers (lymphocytes, mean corpuscular haemoglobin (MCH), mean corpuscular haemoglobin concentration (MCHC), and C-reactive protein (CRP)) were tested between the types of clusters. Due to the nonnormal distribution of the biochemical indicators, a median (Mann-Whitney *U*) test was applied for the statistical analysis. Statistically significant (*p* < 0.05) and borderline statistically significant (*p* < 0.1) results are reported in Table 3.

**Figure 4:**
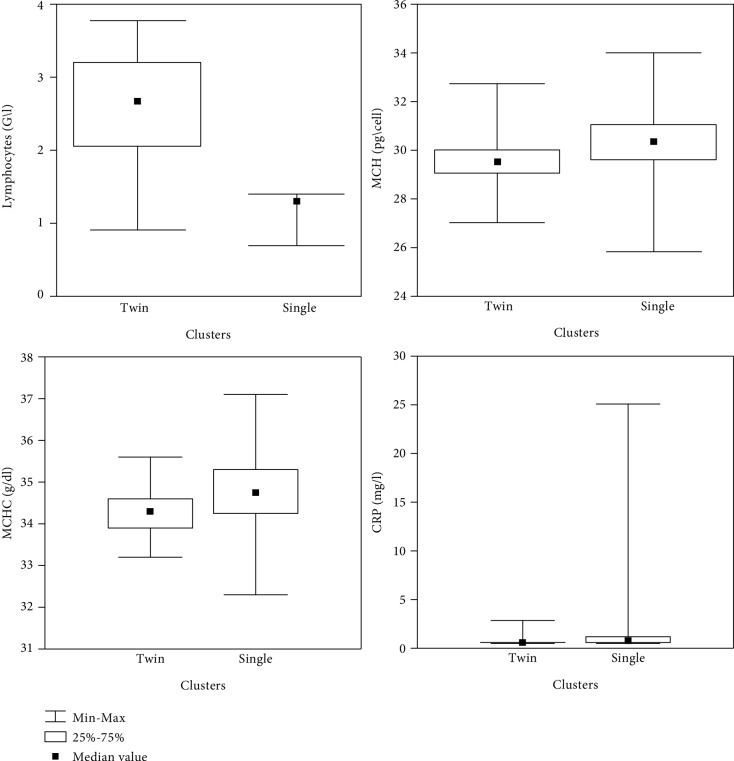
Lymphocytes, MCH, MCHC, and CRP in “twin” (MPV-unrelated) and “single” (MPV-related) patients (*n* = 50).

It can be seen from the results reported in Table 3 that MPV-related pain symptoms have higher MCH, MCHC, and CRP levels than MPV-unrelated pain symptoms. The difference is borderline statistically significant only for lymphocytes. The results are also displayed graphically in Figure 4.

Finally, the specific disease was identified in the patients in the MPV-unrelated and MPV-related groups. Among all pain syndrome patients, eight were diagnosed with a clinical migraine, while the others were diagnosed with other headache syndromes. Surprisingly, seven of the migraine patients belonged to the MPV-related disease group, resulting in a statistically significant difference with the MPV-unrelated group with a *p* value = 0.015 (*χ*^2^ = 5.95, *df* = 1).

## 4. Discussion

Throughout the past decades, mean platelet volume, as a potential marker of platelet reactivity and a surrogate parameter for a broad variety of diseases, has been the subject of numerous investigations. To date, less attention has been paid to neurologic diseases in comparison to cardiovascular diseases [19], cancers [20–26], or genetic issues [27].

Regarding neurologic diseases, we found three studies on fibromyalgia [28–30], which report that the levels of MPV were significantly higher in the fibromyalgia group than in the control group. Such results are not consistent between two chest pain studies for patients diagnosed with acute coronary syndrome (ACS). In a large Chinese study [31], compared with the non-ACS group, the ACS group had significantly higher MPV values, whereas American investigators [32] reported that MPV was not significantly different between ACS-negative and ACS-positive patients. Other examples could easily be found in the literature.

A study conducted by Turkish authors [33] was similar to the investigation reported here. In the Turkish study, MPV was also examined in patients with cerebral venous sinus thrombosis headache admitted to the emergency department. However, no significant difference was determined with primary headache controls, including tension-type headache, migraine, and other types of headache. According to a recent review, several theories about platelet contribution to migraine have been published in the literature. In 1978, a potential relationship between platelets and migraine was proposed. Some authors discussed the interaction with leucocytes, while others targeted serotonin, as platelets contain this neurotransmitter in their dense granules [34]. Indeed, this could be reflected by different mean platelet volumes.

Given our results above, we suggest studying this kind of relationship using an alternative method, such as a taxonomical approach (by dividing the patients into clusters), and not by directly testing only simple independent groups. In our opinion, taxonomic approaches provide much more specific results and more evidence of the similarity and dissimilarity of patients, which could help lead to a hypothesis on MPV-related migraines.

However, a strong limitation of our results is the number of investigated pain syndrome cases (*n* = 50) and the final statistical inference based on the nearly 1% (8/852) of all analysed neurological episodes with diagnosed clinical migraines. It is difficult to obtain more precise statistical data, even though current epidemiological studies estimate the global prevalence of chronic migraine to be approximately 2% of the world population [35]. Moreover, migraine remains undiagnosed and undertreated in at least 50% of patients presenting with headache, and less than 50% of migraine patients consult a physician [36]. Therefore, our findings should be considered with caution. Nevertheless, in our study, the estimated effect of gender confirms the results in the literature that show that migraine affects three times as many women as men [37]. Based on the above findings, we believe that we have made a considerable step forward in migraine research and have provided reliable biomarker references for further studies.

## 5. Conclusions

Based on the literature, the collected study material, and the statistical taxonomical analysis performed here, the following conclusions can be drawn:

1. MPV may implicate a clinical relationship with pain syndromes and migraine episodes
2. Complex taxonomic statistics and clustering data could help analyse data to find new correlations and provide another type of causality
3. We believe that we have been able to determine a credible research pathway for the MPV biomarker assessment of the occurrence of pain syndromes and migraines

## Figures and Tables

**Table 1:** Statistics of the patients diagnosed with neurological disorders according to ICD-10 class.

Disease class	Number	*n*	Fraction
Meningitis	I	10	1%
Neurodegeneration	II	23	3%
Multiple sclerosis	III	10	1%
Epilepsy	IV	420	49%
Pain syndrome	V	50	6%
Stroke	VI	157	18%
Neuralgia	VII	117	14%
Neuropathy	VIII	14	2%
Others	IX	51	6%

**Table 2:** Relative risks for age, sex, MPV, and other biomarkers for pain symptoms (*n* = 50).

Clusters	“Twin” (*n* = 26)		“Single” (*n* = 24)	
Risk factors	RR (95% CI)	*p* value	RR (95% CI)	*p* value
Female vs. male	3.64 (1.46, 9.06)	0.0055	2.44 (1.40, 4.23)	0.0015
Age	0.99 (0.97, 1.01)	0.2740	1.00 (0.99, 1.00)	0.4540
MPV (fL)	0.93 (0.64, 1.37)	0.7290	1.03 (1.02, 1.05)	<0.0001
RBC (mln/mm^3^)	0.74 (0.34, 1.62)	0.4480	0.57 (0.33, 0.96)	0.0363
Haematocrit (%)	0.97 (0.90, 1.05)	0.4530	0.92 (0.86, 0.98)	0.0144
Haemoglobin (g/dL)	0.91 (0.73, 1.14)	0.4190	0.81 (0.68, 0.96)	0.0130

RR: relative risk; CI: confidence interval; MPV: mean platelet volume; RBC: red blood cell.

**Table 3:** “Twin” (*n* = 26) and “single” (*n* = 24) patients' characteristics.

	Mean		SD		Median		Minimum		Maximum		
Clusters	Twin	Single	Twin	Single	Twin	Single	Twin	Single	Twin	Single	*p* value
Lymphocytes (G/l)	2.57	1.13	0.93	0.38	2.67	1.3	0.9	0.69	3.78	1.4	0.0527
MCH (pg/cell)	29.5	30.4	1.29	1.87	29.5	30.4	27	25.8	32.7	34	0.0311
MCHC (g/dL)	34.2	34.7	0.60	1.15	34.3	34.8	33.2	32.3	35.6	37.1	0.0351
CRP (mg/L)	0.22	1.95	0.47	5.64	0.1	0.3	0.0	0.0	2.4	25	0.0171

SD: standard deviation; MCH: mean corpuscular haemoglobin; MCHC: mean corpuscular haemoglobin concentration; CRP: C-reactive protein.

## Data Availability

The data that support the findings of this study are available from the corresponding author upon request.
